# The Contribution of Walking to School in Students' Physical Activity and Its Effect on Being Overweight

**DOI:** 10.1155/2022/2633109

**Published:** 2022-06-06

**Authors:** Hemmat Khodanazari, Abdoul-Ahad Choupani, Iman Aghayan

**Affiliations:** Department of Civil Engineering, Shahrood University of Technology, Shahrood, Iran

## Abstract

**Results:**

The findings of this research showed that being overweight is a concerning issue even in a small-sized and lightly populated city such as Bandar-Turkmen, Iran. Most students (90%) did not perform the required minimum daily activity when commuting to school. Further, overweight could be found even among those students who walked more than one hour to commute to school. The contribution of walking to school to the MVPA of overweight students was found to be low on school days and throughout the year. Counterintuitively, the situation is even worse for nonoverweight students.

**Conclusion:**

The population of overweight students was more active compared to nonoverweight students in general, although they still suffered from excess weight since walking distances were short and not all days were school days. Thus, encouraging students to walk to school is necessary, while it is not sufficient alone as a single measure. Moreover, we recommend that all students should perform extra PA at home, school, or other places, on both school days and all other days of the year.

## 1. Introduction

Children's dependence on motor vehicles has increased in recent decades [1], while the percentage of children walking to school has dropped significantly, especially in wealthy and/or English-speaking countries. For example, 49% of children aged 5–19 commuted to school on foot or by bike in the United States in 1969. However, only 13% of this age group used active modes to travel to school in 2009 [2]. In Switzerland, the proportion of children biking to school has decreased linearly since 1994, mainly in urban areas [3]. Similar declining trends can be observed in other countries such as England [4], Canada (Smart [5]), Australia [6], Portugal [7], Denmark [8], and the Philippines [9]. On the other hand, other countries including Japan [10], Finland [11], and Scotland [12] have been very successful in retaining high levels of children who actively travel to school.

It should be noted that obesity is spreading even among children as the dependence on motor vehicles increases. The World Health Organization (WHO) webpage reported that the number of children and adolescents aged 5–19 who were obese or overweight in 2016 was more than 340 million. Nearly 11% prevalence of obesity at primary school starting age placed Iran, the country under study, on a par with Western countries [13]. More importantly, 77% of overweight children are likely to become overweight adults in the future [14].

According to Mozaffari and Nabaei, being overweight is due to inappropriate diet choices, unhealthy and sedentary lifestyles, and reduced physical activity (PA) [15]. However, active commuting to school (ACS) is one of the great opportunities that can increase energy consumption in students [16]. On these travels, students go to school on foot or by bike.

Some researchers have attempted to summarize and classify the broad effects of transportation, and walking as a healthy travel behavior, on the overall quality of life (QoL) [17]. For example, Dodge et al. classified transportation effects into psychological, physical, and social [18]. Further, Pollard and Lee suggested considering cognitive well-being and economic issues as two further aspects of transportation [19]. Waygood et al. organized the above-mentioned five domains of well-being [20]. They proposed that transport influences QoL in at least three different ways, including access, intrinsic (i.e., during travel), and external (i.e., transport by others).

Various research studies have focused on reasons for the decline in active travels to school. For instance, socioeconomic variables such as higher car ownership [21], higher income [22–24], higher education levels of mothers [25], and unsafe or insecure walking routes [26, 27] can all have a negative influence on students' active travels. Additionally, increase in home-to-school distances is one of the most important factors affecting the reduction in active travels [28]. Similarly, gender impact assessment shows that girls are typically less active than boys in this respect [29].

Some studies consider parents as a significant factor in the behavior of school travel [27]. For example, Park et al. concluded that if the parents walk, their children are also more likely to walk to school [30]. In addition, Mehdizadeh et al. pointed out that parental worries about road safety and kidnapping of their children on the way to school have an influence on reducing students' active travels [31].

The WHO recommends that children and adolescents aged 5–19 years need to accumulate 60 minutes of moderate-to-vigorous PA (MVPA) each day [32]. This requirement determines the duration and intensity of PA for having a positive effect on one's health. Although walking to school makes a meaningful contribution to MVPA for active commuters [12], no study has yet examined whether overweight students who walk to school accumulate the minimum PA and whether inadequate PA is associated with excess weight. It is widely believed that overweight students are less active than their nonoverweight peers [33] because students or their families are more autodependent [21, 34, 35]. Accordingly, this study sought to evaluate whether students' overweight can be attributed to more dependence on motorized modes of travel and the inadequacy of their PA in walking trips. Likewise, the present study attempted to examine whether there is a meaningful difference in prevalence and walking distances among overweight and nonoverweight students. Finally, the study assessed the amount of PA accumulated in school trips and its association with weight status.

Promoting ACS has been the focal point of various research studies and public health policies over the years, if such initiatives significantly increase the MVPA of student populations [36–39]. The MVPA accumulated during active travels is generally considered by research scholars in terms of individual students who actively commute to school while focusing only on education days. However, this perspective places less emphasis on population MVPA since it does not regard the prevalence of walking to school and the fact that students do not go to school on all days of the year. No research has yet studied the contribution of ACS to the MVPA of overweight students and its possible effects on students' weight status. Therefore, evaluating the extent to which future studies and policy interventions should focus on ACS requires a better understanding of the contribution which ACS makes to the MVPA of overweight students. Thus, the main aim of the current study was to conduct a quantitative analysis to assess the contribution of walking to school to the MVPA of overweight students on school days and all days of the year.

Worldwide, a great number of studies have been conducted on the association between weight status and ACS [36–39]. Reviews have shown that there can be a negative or positive or no association between ACS and weight status (Table 1) [37, 39]. Out of the 34 studies conducted on the correlation between ACS and weight status in Table 1, 17 reported no association, and 14 confirmed a negative association. Madsen et al. examined an unexpected positive association between ACS and weight status and found that students who actively commuted to or from the school were more likely to purchase food en route compared to students who traveled by motorized modes [40]. In addition, the positive association between ACS and weight status disappeared when controlled for the confounding effect of purchasing food.

As shown in Table 1, most studies on ACS have been performed in developed countries in North America and Europe, while research regarding students' ACS in middle-income or developing countries in Western Asian or Middle Eastern contexts remains scant [31]. Furthermore, ACS policies such as “Safe Route to School” [41, 42] and “Walking School Bus” [43], which promote children walking collectively to school in an adult-supervised and timetabled manner, have not been implemented in Iran although overweight in childhood is prevalent. This issue and inconsistencies in correlations with ACS make the developing country of Iran an interesting case for further studies. Moreover, the target community of previous studies has been either the girl or boy students of specific educational grades, levels, or school types.

However, the target community of the current study included girls and boys of all 12 grades enrolled in both public and private schools.

The objectives of the current research are as follows:

1. To examine whether overweight students have more dependence on motor vehicles
2. To investigate if students accumulate the minimum PA in school trips
3. To explore the association between the inadequacy of PA in walking trips and excess weight
4. To understand to what extent ACS contributes to the MVPA of overweight students on school days and all days of the year

## 2. Materials and Methods

This study is a cross-sectional evaluation of the differences in the travel patterns of overweight and normal-weight students. The study was aimed at assessing the health implications of walking to school in an Iranian setting. We employed a self-report questionnaire for use in school children aged 7–18 years.

Since the context of the study has an influence on how students commute to school, the city under study, its transportation systems, the educational system, and the types of schools in Iran will be described in the next section.

### 2.1. Context of the Study

The present study focused on the city of Bandar-Turkmen, Golestan, located in Northeastern Iran. Bandar-Turkmen is a coastal city with a moderate and humid climate and an urban population of 53,970 in 2016 [75].

Bandar-Turkmen lacks a regular bus system and shared taxis constitute the only active (para)-transit system. In Iran, the shared taxi system attracts a high modal share and operates similarly to regular bus services in that vehicles are only allowed to follow a specific predetermined path on the network. The service is provided with small-sized vehicles, and the main features are that vehicles only depart if full and that there are no intermediate boarding stops.

School services in Iran are generally provided by minibuses, vans, cars, or taxis, while there are no school buses or minibuses. Additionally, the government does not fund school services, and thus, parents must pay all associated expenses. Hence, there is no distance threshold to be eligible for school services, and students are likely to utilize school services even if their homes are very close to the schools. In addition, the school office may arrange and schedule for minibus or van services if the number of students with the same travel route is high in a school. Otherwise, parents themselves are required to find appropriate shuttle services and reach an agreement with drivers on the prices and schedules.

The educational system in Iran is divided into three main levels of primary, guidance, and high school. All children spend six years of their education at the primary level from the age of six to 12. Further, they attend guidance and high school from the ages of 13 to 15 and 16 to 18, respectively. Schools are single sex in Iran.

The schools in Iran can be broadly classified into two groups, namely, public and private. Public schools are free schools and are administered by government. Private schools charge tuition fees and are administered by private parties. Private schools have higher educational quality than public schools [76].

So far, active travel programs such as “Safe Route to School” [41, 42] and “Walking School Bus” [43] have not been implemented in any of the schools of Bandar-Turkmen.

### 2.2. Questionnaire

Originally, a Norwegian team consisting of researchers and experts in transportation and traffic psychology developed a questionnaire to collect information about the active travels of students [77, 78]. The questionnaire then was translated into English and validated by Nordfjærn et al. [79, 80] and Şimşekoģlu et al. [81].

For the current study, we employed an English version of this questionnaire as a basis, which was then translated into Persian to be used in the present study. The questionnaire employed in the current study was named the active and safe travel to school questionnaire (ASTSQ). Regarding the translation of the ASTSQ into Persian and cultural adaptation, it should be explained that three transportation specialists and three physical activity specialists, who were fluent in both English and Persian, translated the English version of ASTSQ into Persian. Three English language experts then translated the resulting Persian translations into English. Then, by comparing the original English text and the Persian to English translation versions, the necessary corrections were made in the Persian translation. To evaluate the Persian version in terms of cultural phrases and concepts, the Persian questionnaires were sent to 10 faculty members specialized in transportation or physical activity of children and adolescents. They were asked to evaluate the ASTSQ questions and items based on the content validity index (CVI) and score them from one (completely unsatisfactory) to four (completely satisfactory) [82]. If the experts give each question or item a score of three or four, the content validity is satisfactory for that item. According to the experts, changes were made in the travel modes since they differ significantly from country to country. Because the ASTSQ was to be distributed among 23 schools, three students were randomly selected from each school. The ASTSQ was provided to their parents to ensure that they fully understood all aspects of the ASTSQ and had no difficulty understanding its meaning.

The first part of the questionnaire included questions about the socioeconomic characteristics of the household, along with students', fathers', and mothers' travel habits. In the second part of the questionnaire, parents' attitudes toward walking, factors influencing the choice of travel mode, parents' risk assessment of walking, and concerns about active travels to school were investigated.

### 2.3. Sample Size and Sampling Process

The sampling procedure of the current study was a combination of random clustered and stratified sampling techniques. Students were classified based on sex, educational level, and school type, with the number of students in each category following polynomial distribution. Thompson [83] provided a table to estimate the sample size for a polynomially distributed random variables. The sample size increases if the number of variable categories decreases. In this study, sex had the least number of categories and thus was critical for estimating the sample size. Considering that the values of the relative estimation error (*d*) and confidence level (*α*) were selected at 0.05 and 0.01, respectively, the required sample size was 788. Additionally, 1,500 questionnaires, 712 more than the required amount, were distributed among the students according to true population proportions, given that some students would probably not return the questionnaires or that parents would fail to answer the questions completely or fill out the questionnaires inappropriately.

### 2.4. Procedures

#### 2.4.1. Schools' and Students' Recruitment

To select schools, researchers initially consulted with the education authorities of the city, who then, based on their past experiences, recommended inclusion of certain schools due to the close cooperation of their students with survey teams and a high participation rate in past surveys. Similarly, the authorities recommended that specific schools included students of all social classes from all areas, thus making them representative of the entire population of students in the city. A shorter list of schools was prepared from which schools to be included in the study were randomly selected.

The locations of schools in the city of Bandar-Turkmen have generally not been planned with specific geographical or spatial patterns or rules in mind. Public schools can mostly be found in highly populated community areas, whereas private schools tend to be geographically spread around the city. These schools were selected from every area of the city to ensure that all urban regions were covered in terms of socioeconomic characteristics. Likewise, classes within these schools were randomly selected and the students of a class were all given the questionnaires to take home.

The survey was conducted in a total of 23 out of 60 schools in the city, namely, 11, 6, and 6 elementary, guidance, and high schools out of 30, 14, and 16 schools, respectively, in December 2017.

#### 2.4.2. Filling the Questionnaire

School teachers were instructed to ask the students to take the questionnaires home, have their parents, caretakers, or guardians fill them out, and bring them back to schools after three days. Predominantly, 96.3% of parents filled out the forms. The families of students also received written instructions regarding how to fill out the questionnaire. The questionnaire was only filled out for the student who took it home, and the information about other children or students in the household was not collected.

Generally, if the school officials ask the families and students to participate in a survey or complete a questionnaire for a study, the families and students agree and cooperate. Therefore, no written consent was obtained from students and their families to participate in the survey. However, appropriate approvals from officials of the Bandar-Turkmen education bureau and schools were gained prior to the start of the survey. It is important to mention here that gaining approval or permission from an ethics committee for medical experiments or human research is not mandatory in Iran.

#### 2.4.3. Sample Weighting

The total number of enrolled students in the school year 2017–2018 was 10,719, of which 5,651 and 5,068 were boys and girls, respectively. Out of 1,500 distributed questionnaires, students returned a total of 1,072 questionnaires, constituting a response rate of 71.47%, three days after receiving them (Table 2). However, 73 questionnaires were poorly completed, leaving 999 of them remaining for analysis. The final response rate was 66.66%. Table 2 represents the number of students who participated in the survey by sex, educational level, school type, and the number of questionnaires distributed and returned.

**Figure 1:**
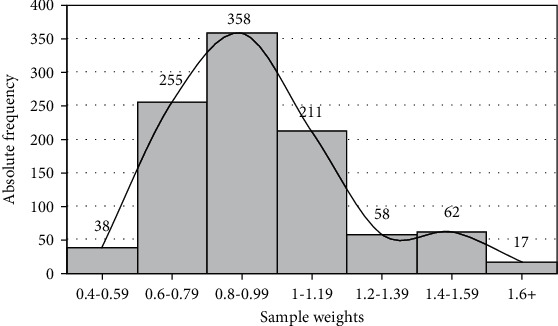
Histogram of sample weights.

Since some questionnaires were not returned in the same proportions as they were distributed, measures had to be taken to reflect the over- or underrepresentation of some groups. Accordingly, sample weights were applied in such a way that the proportions of students were similar in the sample and population in terms of sex, the educational level, and school type. The sum of sample weights was equal to 999 provided that the number of correctly completed questionnaires was 999 (Table 2). Based on the database, public schools had a lower rate of return. Thus, sample weights greater than one were used for public school students.  Figure 1 shows the histogram of the sample weights. The weights of 569 students were in the range of 0.8–1.19 and that the majority of weights were close to one. These weights were applied in analyses presented in the subsequent sections of this study.

Since travel features such as mode and distance did not contribute to sample weighing, it may seem that sample weights are unnecessary for travel analysis and can be excluded from further consideration. However, given that the three sociodemographic variables of sex, educational level, and type of school may indirectly affect travel characteristics, sample weights were applied to implicitly capture the effects of variables on travel patterns.

### 2.5. Measures

The frequency of travel modes and the travel distances were the two measures widely applied in this study. Parents were asked about how many times on school days of the week their children went to school and returned home by each mode of travel. Travel modes were divided into 12 classes in ASTSQ for the convenience of respondents. However, some similar travel modes were aggregated after the data collection process. The four final travel modes were classified as active, when the student went to school and returned home on foot; semiactive, with the student going to school in a motorized vehicle and returning home on foot or vice versa; service, whereby students commuted to school by school services in vehicles which were not owned by the student's family; and motorized where the student commuted to school purely by car, motorbike, taxi, and the like.

The collected questionnaires showed that the changes in travel modes during school days of the week were small. Therefore, we assume that the travel mode is the same in all school days of the week.

Although the current study primarily considered the inclusion of data from students who cycled to or from school, the focus shifted to walking trips because the prevalence of cycling among the students (and residents of the city) was extremely low, and walking was the dominant mode of active traveling. The home-to-school network distance stated by parents in the questionnaires was considered as a proxy for travel distance.

In addition, the body mass index (BMI) was used to determine students' obesity or thinness. The BMI is defined as the body mass divided by height in meters squared (BMI = W/L^2^ kg/m^2^). In the questionnaires, parents were asked to report their children's heights and weights. To determine the weight status of students, *Z*-score graphs, as recommended by the WHO, were separately applied for boys and girls. Using these graphs, the *Z*-score and weight status of each student were determined based on their BMI and age. For example, the student's weight status was considered “normal” if the BMI and age were such that the *Z*-score of the student was between +1 and -2 (Table 3). In this study, the weight status of students was reduced from five to three bins and classified as overweight, normal, or underweight (Table 3). Student weight was deemed above the normal level no matter how overweight he/she was once his/her weight status was overweight.

### 2.6. Methods

In the current study, the prevalence of obesity among students in the small city of Bandar-Turkmen was obtained.

A hypothesis test was conducted to establish whether the proportions of active travels to all made trips were statistically similar between overweight and other weight groups. This test was used two times to compare modal shares between overweight and normal-weight, as well as overweight and underweight students. The null hypothesis was as *p*_1_ = *p*_2_, where *p*_1_ and *p*_2_ were modal shares in the two groups and the alternative hypothesis was *p*_1_ ≠ *p*_2_.

To examine if overweight students make shorter active trips, the distances of active or inactive travels between overweight and normal-weight students were compared. The least significant difference (LSD) test was applied in the analysis of variance (ANOVA). Prior to ANOVA, Levene's homogeneity of variance test was performed to check whether the variances of travel distances were equal across weight groups. The test finding revealed that the variances of travel modes were equal at the *α* = 0.05 significance level. Then, the association analyses between weight status and the distances traveled by different modes of travel were conducted.

The next section investigates whether walking to school can meet the minimum PA requirements recommended by health organizations. The WHO recommends that children and youth aged 5-19 should accumulate at least 60 minutes of MVPA each day [32]. Considering that walking to school makes a meaningful contribution to MVPA [12] and that walking speed is 3.6 km/h, or 1 m/s, for children, the home-to-school distance on active and semiactive travels should be at least 1.8 and 3.6 km, respectively, to ensure that students walk for more than 60 minutes on any given school day.

The research team also evaluated whether students who had enough physical activity on school trips could be overweight. The nonparametric chi-square test was applied to represent the independence of weight status from the adequacy of students' walking distances. In this test, the expected counts of cells in the crosstab of the two nominal variables, i.e., weight status and sufficiency of walking variables, were obtained using an independence assumption, and then, chi-square test was carried out using expected and observed cell counts.

The contribution of walking to school to the MVPA of students was computed. The students do not attend schools on many days of the year, and not all of them walk to school. School days only represent nearly 40% of all days of the year in Iran. Additionally, schools are closed more than four months in a year due to summer and New Year holidays, along with weekends. Therefore, the number of school days attended per year matters, as does active commuting prevalence. The contribution of walking to school to the MVPA of entire population of students was analyzed for school days and all days of the year.

The influence of sex on students' overweight and travel patterns was also investigated.

A logistic regression model was developed to obtain the relation between students' weight and other independent variables affecting it. The response variable was binary, indicating whether the student was overweight or not. In the current research, the number of collected data fields for each student was 130, all of which were considered for incorporation into the logistic model as independent variables. These included the socioeconomic status of the family, characteristics of student's school trips, travel patterns of parents, school features, parents' concerns and perceptions of risk and safety, traffic and geometric risk factors when walking on roads and streets, the importance of factors for parents in selecting travel modes of their children, and parents' attitudes toward walking and were collected and entered in the modeling.

A forward likelihood ratio fitting was used to estimate the parameters. In this method, the variables are added to the model in an order that causes the most significant improvement in the likelihood ratio. The likelihood ratio was defined as the likelihood of the model with more variables (full model) over the likelihood of the model with fewer variables (reduced model).

## 3. Results

### 3.1. Overweight Prevalence

61.98% of students were of normal weight, while 21.76% and 10.33% were overweight and obese, respectively (Table 3). Further, underweight students constituted only a small proportion of the population at 5.9%. In the current study, nearly one-third of students (32.09%) suffered from overweight.

### 3.2. Modal Shares by Weight Status

**Figure 2:**
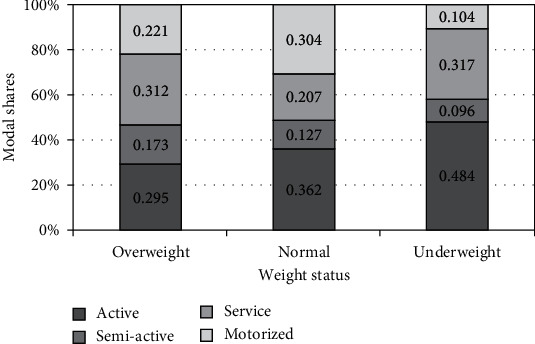
Modal shares by weight status.

Figure 2 shows the modal shares of active and inactive travel modes among students. As can be seen in Figure 2, the sum of the shares related to active and semiactive travel modes of overweight students was approximately 50% and nearly equal to that of normal-weight students.

A hypothesis test was performed to observe whether the proportions of active trips made by the three weight groups were statistically different. The proportions of active trips made by overweight and normal-weight students were not significantly different (*p* value = 0.73), and the same results were obtained when comparing the semiactive trips of the same two groups (*p* value = 0.482) (Table 4).

Investigating the linkage between being overweight and modes of travel using Spearman's rho correlation coefficient demonstrated that there was no statistically significant correlation between these two variables (*ρ* = 0.039 and *p* = 0.001).

This confirmed that active or semiactive travel modal shares were similar for overweight and normal-weight students. Moreover, being overweight and modes of travel were independent of each other.

### 3.3. Average Travel Distances by Mode of Transport

The distances of trips made by students were obtained according to weight class and mode of travel. As represented in Table 5, active and semiactive trips were the shortest, whereby the home-to-school distances were 1.27 and 1.89 km, respectively. Service trips were the longest at 4.77 km. Students often preferred to walk when the home-to-school distance was short. Conversely, when the distance from home to school increased, they walked only one leg of the round trip and used motor vehicles for the other leg. As the distance increased further, parents would take their children to school by privately owned motor vehicles and later pick them up again. However, parents preferred their children to commute to school by services for the longest trips. Therefore, students who traveled with school services had lower incentives to make a modal shift to walking because this would involve distances as long as 4.77 on foot to reach their school. In this regard, students who traveled with motorized modes were more likely to make a modal shift.

ANOVA results using the LSD test demonstrated that distances which overweight students traveled by different modes of travel were similar at the *α* = 0.05 significance level to the distances that nonoverweight students traveled using the same modes.

An association analysis further suggested an insignificant inverse correlation between weight status and the distances of active travels (*ρ* = −0.028 and *p* = 0.130), implying that being overweight was independent of the distances of active travels. As previously mentioned in Modal Shares by Weight Status, we found that being overweightness was irrelevant to travel modes.

The lack of association between weight status and travel modes and distances showed that active travels were not less prevalent among overweight students and that these students were neither less active in school trips compared with other students, nor did they make either fewer or shorter active trips.

### 3.4. Adequacy of PA in Walking to School

This section examines whether walking to school can meet the minimum PA requirements recommended by WHO.  Table 6 shows the frequency of students with sufficient PA in daily school trips.

Out of 293 students who made active travels, the home-to-school distance for 21.16% was ≥1.8 km. In semiactive travels, the home-to-school distance for 16.67% of 120 students was ≥3.6 km. In general, around 20% of students who walked to school performed the minimum required activity in a day. Given that about half of travels were active or semiactive, and around 20% of these met the minimum required PA, only 10% (0.2∗0.5) of all students had enough PA when traveling to school. Most students did not perform the minimum daily activity needed when commuting to school and typical walking distances were not long enough.

One may ask whether excess weight can be found among students who walk sufficiently. Based on the data in Table 7, approximately 30% of students who sufficiently walked on school trips were overweight, while the remaining students were not overweight.

Of the students who did not walk sufficiently on school trips, 30% of them were also overweight, while the remaining students were not overweight. The comparison of column proportions in Table 7 indicates that overweight students were equally distributed among student groups with sufficient and insufficient walking distances.

The nonparametric chi-square test was applied to represent the independence of weight status from the adequacy of students' walking distances. The test finding in Table 7(b) shows that weight status was independent of the sufficiency of walking (*p* value = 0.656). Accordingly, overweight was also found among students who walked sufficiently, and adequacy of walking distances was independent of weight status.

The contribution of walking to school to the MVPA of students was computed as the sum of the walking distances of students with a specific weight status divided by the should-walk distance of the same group (Table 8). The sum of the walking distances of students is a measure of the collective walking distances. In this way, the expression of amount of contribution either in percentage or in minutes of walking can be regarded as a measure of collective walking of students. The should-walk distance of students was computed as the number of students multiplied by 3.6 km. To calculate the average minutes of walking for students, the contribution percentage was multiplied by 60.

Based on data in Table 8, every overweight student walked an average of 39 minutes, while nonoverweight students walked an average of 31 minutes on school trips. To put it differently, walking to or from school accounted for 64.24% and 51.8% of the MVPA of overweight and nonoverweight students, respectively, on schooldays. This again emphasizes that overweight students collectively walked more compared to nonoverweight students.

On a school day, a student who actively commutes to school will walk for an average of 33 minutes regardless of his/her weight status, and he/she performs 55.5% of the minimum MVPA on this trip. Students do not attend schools on many days of the year in Iran, and not all of them walk to school. An active commuter accumulates 13 minutes (22.2%) MVPA when averaged for all days of a year for individuals who walk to or from school. Given that around half of the students do not walk but travel to school by motorized modes, the contribution of walking trips to population MVPA for the entire year further reduces and accounts for only seven minutes a day (11.1%).

### 3.5. Role of Sex and PA

Overweight and obesity prevalence were statistically similar between girls and boys at the *α* = 0.05 level. Overweight girls made fewer and shorter trips compared to overweight boys. In contrast to overweight boys, who walked longer distances than normal-weight boys, the overweight girls' distances of walking were shorter compared with normal-weight girls.

In addition, 20% of all girls undertook the required daily PA when commuting to school, whereas this figure was 21% in boys. In overweight boys, 28.8% performed adequate PA during school trips, but the rest did not perform enough PA. However, only 3.5% of overweight girls performed the minimum PA on school trips.

### 3.6. Logistic Regression

The regression model results and definition of independent variables are shown in Table 9. The logistic regression showed that in the obtained model, parameters such as students' and parents' travel modes, walking duration, and walking distance of students had no significant effect on being overweight, so they were not included in the model. The forward likelihood ratio fitting demonstrated that out of 130 variables that were candidate to be incorporated into model, only the variables presented in Table 9 had a statistically significant relationship with being overweight and the rest of variables were excluded from model by the fitting strategy.

The four variables of student grade, travel mode of student's father, importance of travel safety, and cost for parents were parameters that had a significant effect at *α* = 0.05 on being overweight. In Table 9, the ModeSafe and ModeCost variables show to what extent safety and costs matter to parents when they select the means of transport their children should travel to school with. As can be seen in Table 9, if the father of the student used motor vehicles for his own travels, the probability of the student becoming overweight increased. Moreover, as the student grade increased, this probability decreased.

In previous sections, it was concluded that overweight students do not have more dependence on motor vehicles, and students' being overweight is irrelevant to the mode of travel and walking distance. The logistic regression modeling led to the same result, and parameters such as travel mode, walking distance, and travel distances of students had no influence on overweight. Logistic regression also suggested that the way parents' attitudes toward the choice of travel mode affects overweight in their children is not clear.

## 4. Discussion

In the current study, around a third of students (32.09%) suffered from overweight, which is a concerning issue in Bandar-Turkmen. This is consistent with the findings of Nabavi et al., who demonstrated 33.1% obesity for students aged 7 to 12 years in Semnan, Iran [84]. Similar values have been reported for other highly populated cities in Iran [15, 85], the US (OECD, 2017), and China [86]. The prevalence of overweight in the small-sized and sparsely populated city of Bandar-Turkmen was as high as other densely populated cities in Iran as well as other countries around the world.

The findings of the present study revealed that students' overweight was irrelevant to walking to school. Moreover, the amount of physical activity performed on school trips was very low. Thus, programs and interventions for PA promotion should encourage students and their families to increase their children's PA not only when commuting to school but also at other times and places and on all days of the year.

As previously mentioned in Average Travel Distances by Mode of Transport, the average walking distances of overweight students were like those of nonoverweight students. However, the measure for collective walking of students revealed that overweight students walk more than their normal-weight peers. This demonstrates that applying measures of individual walking would lead to misleading data. It was better to apply indicators that consider overall population walking. In the current study, the individual perspective resulted in an underestimation of the MVPA of overweight students.

The contribution of walking to school to the MVPA levels of overweight students was larger compared to nonoverweight students. However, the amount of contribution of walking to school to the MVPA of overweight students was low both on school days and throughout the year. The situation is even worse for nonoverweight students.

Although parental attitudes toward selecting travel modes were found to be significant for their children's overweight [41], the developed logistic regression model in this study showed that the effect of parental attitudes does not match subjective realities. If walking is taken into consideration as a travel mode with the lowest cost, parents more concerned about costs should tend to want their children to walk more. If children were then to meet their parents' expectations and walk to school, resulting in weight reduction, the probability of being overweight would be expected to decrease. However, the logistic model shows the opposite. Hence, one can conclude that the importance of mode cost should not be included in the model since its sign is not logical, or that if this relation is positive, another mechanism exists for the effect of mode cost on overweightness which cannot be captured by the logistic regression.

It is widely believed that overweight students are less active than their nonoverweight counterparts [33]. However, in the current study, overweight students were not found to be less physically active than nonoverweight students when traveling to school.

Measuring the energy expenditure of obese boys at home, school, and outside home, Waxman and Stunkard concluded that there was no significant difference in calorie expenditure between obese and nonobese boys at home and that obese boys had greater energy consumption than nonobese ones outside home and school [87]. This is in line with the findings of the current study indicating that overweight students were not less active in school trips.

Hatami et al. confirmed that dietary factors including higher energy intake and consumption of low rates of fruits and vegetables were the main determinants of overweight and obesity among Iranian students [88]. Therefore, it can be implied that greater food intake or inactivity at home or outside the school may be associated with, or the cause of, overweight.

Lowry et al. remarked that combining PA with a reduced fat and calorie diet should receive greater attention to achieve a healthier weight [89]. Wu et al. investigated the long-term effectiveness of diet-plus-exercise interventions vs. diet-only interventions for weight loss and concluded that a combined diet-plus-exercise program provided greater long-term weight loss than a diet-only program [90].

The authors speculate that two solutions could be effective in achieving a healthy weight and increasing the physical activity of students. But these solutions and their impact on body weight status need to be investigated for an Iranian setting.

The first solution is to encourage families to increase their children's PA not only when commuting to school but also at other times and places and on all days of the year. In line with this recommendation, the future researches need to measure the daily physical activity of students and they should not focus only on active trips to school. Students who travel to school via motorized modes may perform PA at home, parks, play grounds, gyms, etc. Therefore, measuring the physical activity throughout the day is recommended.

The second solution is to implement diet-plus-exercise intervention among the school students. Current study only analyzed the PA of students when commuting to school, and it did not evaluate the diet and nutritional status of the school students. To the best of our knowledge, other local studies have not examined the effectiveness of diet-plus-exercise interventions yet. The local researches studied the diet and exercise interventions for weight control separately. But since the previous experience in other countries show that diet-plus-exercise interventions were more successful than diet-only programs, the authors speculate the diet-plus-exercise intervention will also be more effective than diet-only interventions for weight loss in an Iranian context. But, this needs to be confirmed for an Iranian setting by further studies.

## 5. Limitations of the Study

The data required for this research were collected using a self-report questionnaire. One of the limitations of the current study was that parents report the height and weight of their school children. Many studies warn about the parents' perceptions of their school children's weight status [91, 92]. Adolescent weight status was accurately assessed by 60% of mothers, underestimated by 35% of mothers, and overestimated by 5% of mothers [91]. It is sophisticated to measure students' height and weight using measurement tools to avoid inaccurate parents' perceptions of their children's weight status.

The current study focused on the health implications of doing physical activity during school trips. But some students who commute to school by motor vehicles may perform physical activities at home or school, or in other places like playgrounds, parks, or gyms. So, adopting a holistic approach and measuring the physical activity throughout the day is recommended. It is also strongly suggested to quantify the students' physical activity on weekends or other holidays in a year when students do not go to school.

The research team relied on association analysis using cross-sectional data. But instead of conducting association analyses and developing logistic regression models, exploring the cause-and-effect relations between overweight and other factors would be more helpful. For this purpose, we recommend developing structural equation models (SEMs). SEMs show the complex interaction system between the overweight and other explanatory variables and the cause-and-effect relationship between them via a system of equations. In this equation system, a network of cause-and-effect relationship is established. Extracting this set of relationships between all variables is more robust and helpful than understanding the association between two variables or a regression function among a limited number of variables. The other reason for suggesting structural equations is that even though numerous variables were tried for inclusion in the logistic regression models, a few of them entered the model, which may be due to not capturing the systematic relation between variables.

## 6. Conclusion

This study was defined to investigate the attachment of overweight students to motor vehicles on school trips. For this purpose, the prevalence of active trips and the distance traveled by different means of travel was compared between the overweight and nonoverweight students. This article also appraised what percentage of students perform the minimum amount of PA recommended by the WHO. Exploring the linkage between the inadequacy of PA in walking trips and excess weight was another goal of the study. The contribution of walking to school to the MVPA of students on school days and all days of the year was also examined.

Neither overweight students nor their parents were more attached to motor vehicles in school trips. Walking trips were not less prevalent among overweight students and the average walking distances of overweight students were not shorter than those of nonoverweight students. Being overweight was not associated with travel modes or distances.

Only 10% of students walked more than one hour on school days, thus performing the minimum required activity when commuting to school. The remaining students would have to perform physical activities at home or school or other places like playgrounds to satisfy this requirement. Although some students walked more than 60 minutes and 3.6 km or more every school day, they were still overweight.

The contribution of walking to school to population MVPA throughout the year was extremely low, probably because of short and inadequate walking distances and the fact that not many days are considered as school days. The contribution of walking to school to the MVPA of overweight students was possibly more than that of nonoverweight students. Parents' attitudes toward the safety and cost of their children's travel mode did affect overweight, but it is not clear how exactly.

It is recommended that local health and transportation policymakers should not only rely on developing walking trips. Instead, they should implement programs to encourage students to build healthy eating habits, alongside finding ways for PA promotion.

## Figures and Tables

**Table 1:** The association between ACS and weight status in previous studies^∗^.

Continent	Negative association	No association	Positive association	Total
North America	(6) [44] [45] [46] [47] [48] [49]	(4) [50] [51] [52] [53]	(2) [54] [40]	(12)
South America	(2) [55] [56]	(0)	(0)	(2)
Europe	(3) [57] [58] [59]	(10) [60] [61] [62] [63] [64] [65] [66] [67] [7] [68]	(1) [69]	(14)
Australia	(1) [70]	(3) [71] [72] [73]	(0)	(4)
Asia	(2) [74] [9]	(0)	(0)	(2)
Total	(14)	(17)	(3)	(34)

^∗^The numbers in parentheses show the number of studies in the category.

**Table 2:** Populations and samples, with distributed and used questionnaire counts.

Education level	Population count	# Distributed questionnaires	# Used questionnaires	Sex	Population count	# Distributed questionnaires	# Used questionnaires
Primary school	6,021	840	628 (74.76%)	Male	3,138	437	306 (70.02%)
				Female	2,883	403	322 (79.90%)
Guidance school	2,488	345	188 (54.49%)	Male	1,342	186	96 (51.61%)
				Female	1,146	159	92 (57.86%)
High school	2,210	315	183 (58.10%)	Male	1,171	167	124 (74%)
				Female	1,039	148	59 (39.86%)
Total	10,719	1,500	999 (66.6%)		10,719	1,500	999

# = number of.

**Table 3:** *Z*-score ranges and students' weight stat.

Weight status (5 bins)	*Z*-score	# Students	Percent (%)	Weight status (3 bins)	# Students	Percent (%)
Obese	>+2	94	10.33	Overweight	292	32.09
Overweight	Between +1 and +2	198	21.76			
Normal	Between -2 and +1	564	61.98	Normal	564	61.98
Thin	Between -3 and -2	33	3.63	Underweight	54	5.93
Severely thin	<-3	21	2.31			

# = number.

**Table 4:** Equality of modal shares for overweight and normal/underweight students, *p* value, and hypothesis test result.

Weight status	Travel mode			
	Active	Semiactive	Motorized	Service
Normal	0.73 (accept)	0.482 (accept)	0.004 (reject)	0.013 (reject)
Underweight	0.031 (reject)	0.201 (accept)	(accept)	1 (accept)

The numbers represent the *p* value of the tests. The null hypothesis accept/reject result (at *α* = 0.05) is shown in parentheses.

**Table 5:** Average home-to-school distance by weight status and mode of travel.

Mode	Active			Semiactive			Motorized			Service		
Weight status	Sample size	Distance mean	Distance stdev	Sample size	Distance mean	Distance stdev	Sample size	Distance mean	Distance stdev	Sample size	Distance mean	Distance stdev
Overweight	78	1.58	3.22	45	2.05	2.83	57	2.53	3.10	75	4.70	4.90
Normal	189	1.08	1.65	71	1.85	1.90	168	3.54	4.30	107	4.74	7.15
Underweight	25	1.71	1.52	4	0.69	0.347	5	1.49	2.14	17	5.23	6.73
Total	292	1.27	2.18	120	1.89	2.27	230	3.25	4.02	199	4.77	6.33

stdev = standard deviation.

**Table 6:** Frequency of students with sufficient PA in daily school trips.

Travel	Home-to-school distance (km)	Absolute frequency				Relative frequency			
		Overweight	Normal	Underweight	Total	Overweight	Normal	Underweight	Total
Active	<1.8	62	157	12	231	78.48	83.07	48	78.84
	≥1.8	17	32	13	62	21.52	16.93	52	21.16
	Total	79	189	25	293	100	100	100	100
Semiactive	<3.6	36	60	4	100	80	84.51	100	83.33
	≥3.6	9	11	0	20	20	15.49	0	16.67
	Total	45	71	4	120	100	100	100	100

**Table:** (a) Cross-tabulation

			Is walking distance adequate?		
			Yes	No	Total
Overweight	Yes	Count	26	229	255
		% With adequacy of walking	32.5	31.1	30.33
	No	Count	54	532	586
		% With adequacy of walking	67.5	69.9	69.67
Total		Count	80	761	841

**Table:** (b) Association tests

Test	Value of statistics	df	*p* value (2-sided)
Pearson chi-square	0.199	1	0.656
Likelihood ratio (*G*-test)	0.196	1	0.658
Linear-by-linear association	0.198	1	0.656

df = degree of freedom.

**Table 8:** Contribution of walking to school to daily MVPA in students who commute to school actively.

Weight status	Mode	# Students	Walking distance of school trips (km)	Should-walk distance (km)	% Contribution (minutes of walking)	
					School days	All days
Overweight	Active	77	189.94	277.3	68.49 (41)	27.4 (16)
	Semiactive	45	92.26	162	56.97 (34)	22.8 (14)
	Total	122	282.20	439.3	64.24 (39)	25.7 (15)
Nonoverweight	Active	214	459.79	769.9	59.72 (36)	23.9 (14)
	Semiactive	75	78.54	269.3	29.16 (17)	11.7 (7)
	Total	289	538.33	1,039.2	51.80 (31)	20.7 (12)
Total	Active	291	650	1,047	62.04 (37)	24.82 (15)
	Semiactive	120	171	431	39.60 (24)	15.84 (10)
	Total	411	821	1,478	55.50 (33)	22.20 (13)

**(a):** 

Variable	Coefficient	Standard error	Wald	*p* value	Exponent
Grade	-.149	.019	60.773	.000	0.861
FMode	0.119	.050	5.625	.018	1.127
ModeSafe	-.209	.073	8.250	.004	1.233
ModeCost	0.110	.048	5.293	.021	.895

**(b):** 

Variable	Description
Is overweight	0-no, 1-yes
Grade	Student grade: 1–12
FMode	Travel mode of student's father in his own travels: 1-active, 2-inactive
ModeSafe	In selecting your child's travel mode on school trips, how important is the travel safety to you? 1, unimportant; 2, little; 3, moderate; 4, much; and 5, very much
ModeCost	In selecting your child's travel mode on school trips, how important is the travel cost to you? 1, unimportant; 2, little; 3, moderate; 4, much; and 5, very much

## Data Availability

The data used to support the findings of this study cannot be made available due to privacy and legal concerns.

## Additional Points

*Code Availability (Software Application or Custom Code).* Code sharing is not applicable to this article as no codes were generated during the current study.
